# Receptor Heterodimerization and Co-Receptor Engagement in TLR2 Activation Induced by MIC1 and MIC4 from *Toxoplasma gondii*

**DOI:** 10.3390/ijms20205001

**Published:** 2019-10-10

**Authors:** Flávia Costa Mendonça-Natividade, Carla Duque Lopes, Rafael Ricci-Azevedo, Aline Sardinha-Silva, Camila Figueiredo Pinzan, Ana Claudia Paiva Alegre-Maller, Lilian L. Nohara, Alan B. Carneiro, Ademilson Panunto-Castelo, Igor C. Almeida, Maria Cristina Roque-Barreira

**Affiliations:** 1 Laboratory of Immunochemistry and Glycobiology, Department of Cell and Molecular Biology and Pathogenic Bioagents, Ribeirão Preto Medical School, University of São Paulo (FMRP/USP), Ribeirão Preto SP 14049-900, Brazil; flavia.bioquimica@usp.br (F.C.M.-N.); cduquelopes@gmail.com (C.D.L.); rrazevedo@gmail.com (R.R.-A.); aline.sardinhadasilva@nih.gov (A.S.-S.); capinzan@gmail.com (C.F.P.); anapaiva@fag.edu.br (A.C.P.A.-M.); 2 Border Biomedical Research Center (BBRC), Department of Biological Sciences, University of Texas at El Paso (UTEP), El Paso, TX 79968, USA; llnohara@msl.ubc.ca (L.L.N.); alan.fiocruz@gmail.com (A.B.C.); 3 Institute of Medical Biochemistry, Program of Molecular Biology and Biotechnology at Federal University of Rio de Janeiro (UFRJ), Rio de Janeiro RJ 21941-599, Brazil; 4 Department of Biology, Faculty of Philosophy, Sciences and Letters at Ribeirão Preto, University of São Paulo USP (FFCLRP/USP), Ribeirão Preto SP 14040-900, Brazil; apcastelo@usp.br

**Keywords:** *Toxoplasma gondii*, microneme proteins, Toll-like receptor 2, TLR co-receptors, TLR heterodimerization, CD14, CD36

## Abstract

The microneme organelles of *Toxoplasma gondii* tachyzoites release protein complexes (MICs), including one composed of the transmembrane protein MIC6 plus MIC1 and MIC4. In this complex, carbohydrate recognition domains of MIC1 and MIC4 are exposed and interact with terminal sialic acid and galactose residues, respectively, of host cell glycans. Recently, we demonstrated that MIC1 and MIC4 binding to the N-glycans of Toll-like receptor (TLR) 2 and TLR4 on phagocytes triggers cell activation and pro-inflammatory cytokine production. Herein, we investigated the requirement for TLR2 heterodimerization and co-receptors in MIC-induced responses, as well as the signaling molecules involved. We used MICs to stimulate macrophages and HEK293T cells transfected with TLR2 and TLR1 or TLR6, both with or without the co-receptors CD14 and CD36. Then, the cell responses were analyzed, including nuclear factor-kappa B (NF-κB) activation and cytokine production, which showed that (1) only TLR2, among the studied factors, is crucial for MIC-induced cell activation; (2) TLR2 heterodimerization augments, but is not critical for, activation; (3) CD14 and CD36 enhance the response to MIC stimulus; and (4) MICs activate cells through a transforming growth factor beta-activated kinase 1 (TAK1)-, mammalian p38 mitogen-activated protein kinase (p38)-, and NF-κB-dependent pathway. Remarkably, among the studied factors, the interaction of MIC1 and MIC4 with TLR2 *N*-glycans is sufficient to induce cell activation, which promotes host protection against *T. gondii* infection.

## 1. Introduction

*Toxoplasma gondii*, a ubiquitous intracellular protozoan parasite of the phylum Apicomplexa, has received considerable scientific and medical attention due to its high transmissibility and endemicity [1]. It has been estimated that approximately 30% of the human population is infected by *T. gondii* [2,3]. Although *T. gondii* infection is typically asymptomatic in healthy individuals, it often causes severe disease in fetuses and immunocompromised individuals [4]. Host cell invasion by *T. gondii* is an active process that relies on the motility of the tachyzoite, which requires its actomyosin system, and protein secretion from two apical organelles, micronemes and rhoptries [5]. Some microneme proteins (MICs) are secreted as complexes, such as those formed by MIC1, MIC4, and MIC6. MIC1 and MIC4 are exposed on the tachyzoite surface and bind to host cell surface receptors, and MIC6 is a transmembrane protein that binds the complex to the parasite surface. Together, these proteins promote tachyzoite adhesion and subsequent host cell invasion [6,7,8].

Host cell adhesion and invasion by *T. gondii* occurs with contributions of carbohydrate recognition [9,10,11], and it is known that MIC1 and MIC4 include carbohydrate recognition domains (CRD) [12,13,14]. MIC1 interacts with the terminal α(2-3)-sialyl residue linked to β-galactoside [8,15,16], and MIC4 interacts with terminal β(1–4)- or β(1–3)-galactose residues [6,14,16].

Interactions with MIC1 and MIC4 activate immune cells [16,17,18], as was first shown by our previous finding that a lactose-binding fraction (Lac+) of soluble *T. gondii* antigens, which contains MIC1 and MIC4, stimulates adherent mouse spleen cells to produce seven-fold higher levels of IL-12 than unstimulated control cells [17]. In addition, immunization of mice with Lac+, recombinant microneme protein (rMIC) 1, or rMIC4 conferred protection against *T. gondii* infection [17,18]. Pro-inflammatory cytokines are frequently produced in response to the interaction of pattern recognition receptors (PRRs) with pathogen-associated molecular patterns (PAMPs), followed by cell signaling [19]. The best-characterized PRRs are the Toll-like receptors [20,21], which signal via a pathway that is dependent on the adaptor protein MyD88 [22] and components of the post-receptor signaling cascade responsible for nuclear factor-kappa B (NF-κB) activation [20]. This explains the high susceptibility of MyD88-knockout mice to *T. gondii* infection and suggests that TLRs play a fundamental role in recognizing parasite components [23] and triggering the innate immune response. Nonetheless, so far only a few *T. gondii* components have been identified as TLRs agonists: (*i*) Profilin, a ligand of murine TLR11 [24,25], TLR12 [24,26], and human TLR5 [27]; (*ii*) glycosylphosphatidylinositol (GPI)-anchored proteins, which activate TLR2 and TLR4 [28]; and (*iii*) parasite nucleic acids, which interact with TLR7 and TLR9 [24,29]. Recently, we reported that rMIC1 and rMIC4 binding to TLR2 and TLR4 on phagocytes up-regulated IL-12 production [16]. Focusing on the interactions between MICs and TLR2, we found that rMIC1 recognizes the second, third, and fourth N-glycans of the receptor, while rMIC4 binds only to the third TLR2 N-glycan. Studies on the interaction of TLR2 with lipopeptide agonists showed that TLR2 heterodimerization with TLR1 or TLR6 amplifies the range of possible TLR2 agonists [30], and engagement of the co-receptors CD14 (a GPI-anchored protein) and CD36 (a class B scavenger receptor) increases TLR2-triggered cell signaling [31,32,33].

In the current study, we investigated analogous aspects of TLR2 interactions with rMICs and found that among the studied factors, only TLR2 is crucial for cell activation. Although receptor heterodimerization and co-receptor engagement increase rMIC-induced cell activation, they are not required. We also identified the signaling proteins involved in the process stimulated by rMICs that culminates in inflammatory cytokine production.

## 2. Results

### 2.1. IL-12 Production in rMIC1- or rMIC4-Stimulated Macrophages Is Dependent on TAK1, p38, and NF-κB Phosphorylation

**Figure 1:**
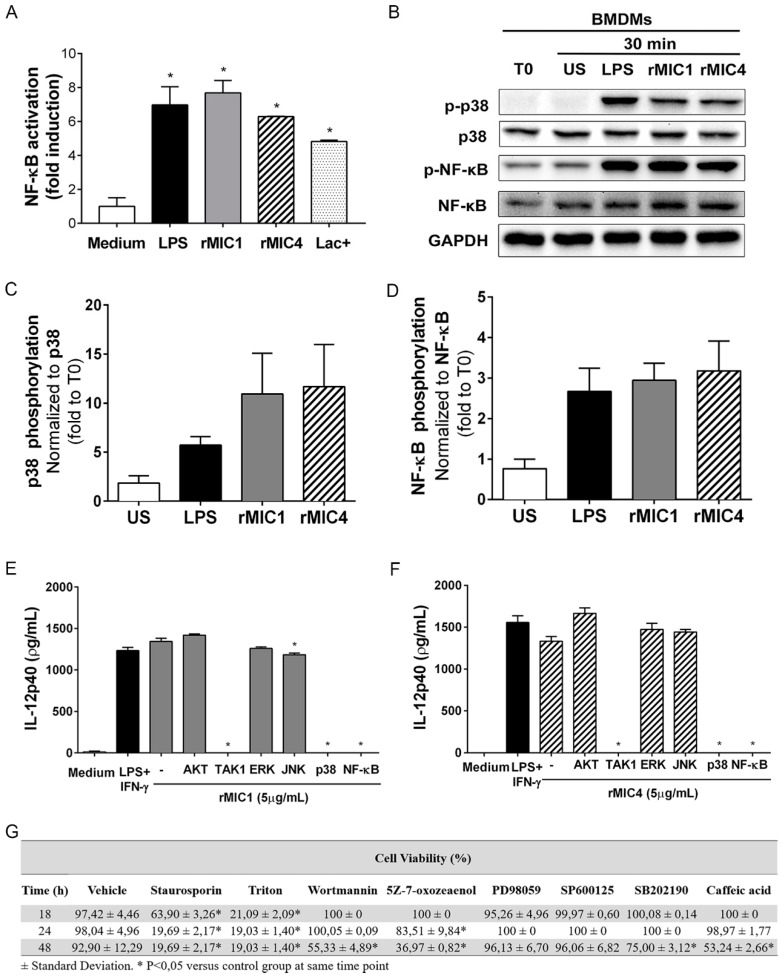
rMIC1 and rMIC4 induce IL-12 production by macrophages through activation of TGF-β activated kinase 1 (TAK1), p38, and nuclear factor-kappa B (NF-κB). (**A**) RAW264.7-*luc* murine macrophages were stimulated with the following preparations of *Toxoplasma gondii* microneme proteins: rMIC1 (5 µg/mL), rMIC4 (5 µg/mL), and the native Lac+ fraction (5 µg/mL), which contains MIC1 and MIC4. LPS (500 ng/mL) and medium were used as the positive and negative controls, respectively. Cells were lysed 4 h post-stimulation, and NF-κB activation was inferred from the luminescence measurements. Data are expressed as the mean ± SD of triplicate wells, and data from three independent experiments yielding similar results. (**B**) Total lysates of bone marrow-derived macrophages (BMDMs) was were 30 min after stimulation with rMIC1 (5 µg/mL), rMIC4 (5 µg/mL), or LPS (1 µg/mL). As controls, cells were also incubated with medium (i.e., unstimulated cells [US]) or sampled at time zero (i.e., T0), as indicated at the top of the panels. Immunoblotting was performed to assess total p38 and the p65 subunit of NF-κB, as well as their phosphorylated forms (p-p38 and p-NF-κB). Glyceraldehyde-3-phosphate dehydrogenase (GAPDH) was used as a loading control. (**C**,**D**) Densitometric analysis to quantify the p38 and NF-κB bands in the Western blot (panel B). (**E**,**F**) IL-12 concentrations in the supernatant of BMDM cultures that were pretreated with vehicle (-) or pharmacological inhibitors of AKT (Wortmannin, 100 nM), TAK1 (5Z-7-oxozeaenol, 100 nM), ERK (PD9805, 20 μM), JNK (SP600125, 20 μM), p38 (SB202190, 20 μM), or NF-κB (Caffeic acid, 15 µg/mL) for 3 h, and subsequently stimulated with (**E**) rMIC1 (5 µg/mL) or (**F**) rMIC4 (5 µg/mL) for 24 h as assessed by ELISA. LPS (1 µg/mL) and medium were used as the positive and negative controls, respectively. (**G**) Viability of BMDMs pretreated with pharmacological inhibitors of signaling molecules. Statistical analysis comparing the viability of cells (%) that were pretreated with each inhibitor and stimulated with rMIC1 or rMIC4 to that of cells stimulated with rMICs but not pretreated with pharmacological inhibitors (vehicle). Staurosporine (2 µM) and Triton X-100 (1% *v*/*v*) were used to induce in vitro apoptosis. The data are from two independent experiments yielding similar results. (*) *p* < 0.05 by one-way ANOVA, followed by Bonferroni’s post-test.

Recently, we demonstrated that, by interacting with the N-linked glycans of the TLR2 and TLR4 ectodomains, the rMIC1 and rMIC4 proteins from *T. gondii* induce macrophages and dendritic cells to produce pro-inflammatory cytokines, such as IL-12, TNF-α, and IL-6, through MyD88-dependent NF-κB activation [16]. Here, we addressed which downstream signal transduction pathways are involved in the cell response to rMIC1 or rMIC4. The preparations that were assayed for their ability to stimulate cell activation were the recombinant microneme proteins rMIC1 and rMIC4 and the Lac+ fraction, which is a tachyzoite fraction containing soluble antigens, including native MIC1 and MIC4, that was obtained by affinity binding to immobilized lactose. As shown in Figure 1A, after 2 h of stimulation with rMIC1, rMIC4, or Lac+, RAW264.7-*luc* macrophages displayed NF-κB activation, with an intensity comparable to that induced by LPS, which was used as a positive control. We also assayed the ability of rMIC1 and rMIC4 to stimulate bone marrow-derived macrophages (BMDMs) from C57BL/6 mice, a cell preparation that was differentiated in vitro in the presence of granulocyte/macrophage colony-stimulating factor [34] and verified to express (at 95.3%) F4/80 (Figure S1). Under both stimuli, p38 and NF-κB phosphorylation levels (Figure 1B–D) were 11- and three-fold higher, respectively, than the basal levels in unstimulated control cells at time zero. These results showed that native and recombinant microneme proteins can activate macrophages through signaling pathways that involve NF-κB and p38. We then examined the proteins involved in the downstream signaling pathways for BMDM activation, as measured by IL-12 production. Then, we either pretreated or not BMDMs with pharmacological inhibitors of Ser/Thr kinase (AKT) (wortmannin), TAK1 (5Z-7-oxozeanol), extracellular signal-regulated protein kinase (ERK1/2) (PD98059), c-Jun N-terminal kinase (JNK) (SP600125), p38 (SB202190), and NF-κB (caffeic acid). Next, we stimulated the cells with rMIC1 (Figure 1E) or rMIC4 (Figure 1F). The IL-12 levels produced by untreated BMDMs that were stimulated with microneme proteins were close to those in LPS-stimulated BMDMs (the positive control). These levels were maintained in BMDMs pretreated with the AKT, ERK1/2, and JNK inhibitors. In contrast, IL-12 production stimulated by rMIC1 or rMIC4 was blocked in BMDMs pretreated with an inhibitor of TAK1, p38, or NF-κB. To validate the assay, especially regarding possible bias due to a potential cytotoxic effect of the inhibitors, we compared the viability of rMIC-stimulated and inhibitor-pretreated BMDMs to that of rMIC-stimulated and non-pretreated BMDMs (preincubated with vehicle). The cells displayed similar viability at 24 h, regardless of inhibitor pretreatment or not (Figure 1G). Thus, the BMDMs remained viable despite pretreatment with the pharmacological inhibitors. The results shown in Figure 1 indicate that IL-12 up-regulation in rMIC1- or rMIC4-stimulated cells occurs in a manner that is critically dependent on TAK1, p38, and NF-κB activation.

### 2.2. TLR2 Is Required for rMIC1- or rMIC4-Triggered Cell Activation, and Heterodimer Formation and Co-Receptor Engagement Enhance the Cell Response

**Figure 2:**
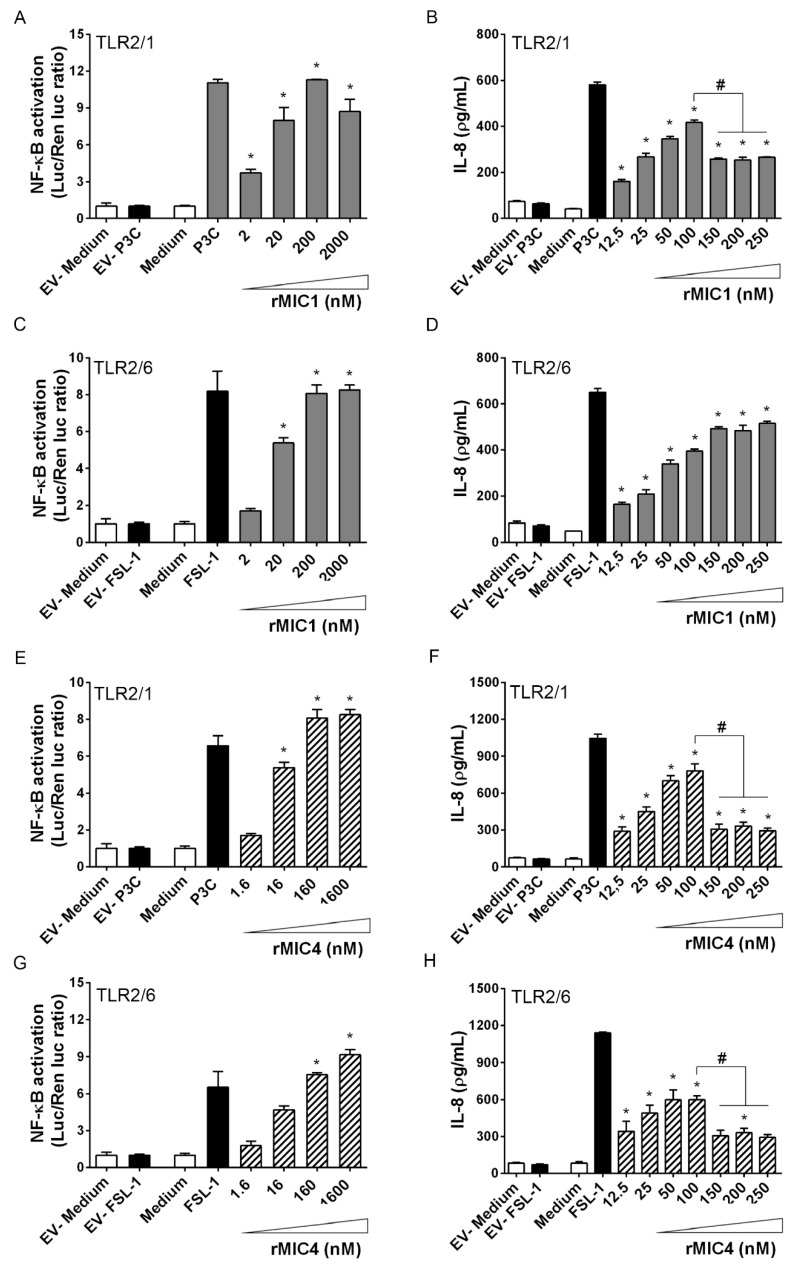
TLR2 is required for in vitro cell activation induced by microneme proteins. HEK293T cells were transfected with CD14, CD36; a pair of TLRs (either TLR2/1 or TLR2/6); and an NF-κB-dependent luciferase reporter (pELAM-firefly luciferase) and a constitutive Renilla luciferase reporter construct (internal control). We maintained a constant amount of DNA in each transfection by adding an empty expression vector. After 48 h of transfection, we stimulated the cells with the following agonists: Pam3CSK4 (P3C, 1 nM) for cells transfected with TLR2/1 (**A**,**B**,**E**,**F**) and FSL-1 (1 nM) for cells transfected with TLR2/6 (**C**,**D**,**G**,**H**). Medium and an empty vector (stimulated with medium or an agonist) were used as negative controls for cell stimulation. Different concentrations of rMIC1 (**A**–**D**) or rMIC4 (**E**–**H**) (indicated on the coordinate axis) were used to stimulate the transfected cells. Cells were lysed at 4 h post-stimulation, and luminescence intensity was measured to assess NF-κB activation. The IL-8 concentration was measured in cell supernatants harvested at 24 h post-stimulation. The data are from three independent experiments yielding similar results. Statistical differences were determined by (*) comparing the responses of cells stimulated with microneme proteins to the responses of unstimulated cells (medium, negative control). In addition, statistical comparisons (#) of responses elicited by 100 nM of rMIC proteins versus the response in the 150, 200, and 250 nM of rMIC were also performed. (* and #) *p* < 0.05 by one-way ANOVA followed by Bonferroni’s post-test.

As previously demonstrated, rMIC1 and rMIC4 interact with TLR2 on transfected HEK293T cells [16]. Because TLR2 molecules are expressed on the cell surface as heterodimers with TLR1 or TLR6 [20], we assessed whether cell activation stimulated by microneme proteins requires receptor heterodimerization. We transfected HEK293T cells with either TLR2 alone or with either TLR1 or TLR6 to form TLR2/1 or TLR2/6 heterodimers. The cells were also co-transfected with one of the co-receptors CD14 and CD36 or both CD14 and CD36, along with the NF-κB-dependent ELAM-luciferase reporter gene construct. Both the luciferase assay for the detection of NF-κB activation (Figure 2A,C,E,G) and the IL-8 concentration in the supernatants of HEK293T cultures (Figure 2B,D,F,H) showed cell activation. Different concentrations of rMIC1 or rMIC4 stimulated TLR2/1- and TLR2/6-transfected cells and resulted in a dose-dependent increase in NF-κB activation and IL-8 production, and both results indicated cell activation. Activation of TLR2/1-expressing cells with rMIC1 or rMIC4 and activation of TLR2/6-expressing cells with rMIC4 showed a bell-shaped curve as a function of dose (Figure 2B,F,H). Maximum IL-8 production was induced by 100 nM rMIC and was close to the amount induced by the positive control agonists P3C (for TLR2/1) and FSL-1 (for TLR2/6). Activity increased dose-dependently at 10–100 nM microneme protein, but decreased at higher tested concentrations (150, 200, and 250 nM). The observed reduction in activity at higher concentrations of microneme protein may be due to a cytotoxic effect of the rMIC, especially rMIC4. Microneme protein concentrations as low as 12.5 nM were sufficient to induce significantly higher responses than those in the negative controls. Our results indicate that rMIC1 and rMIC4 interact with both the TLR2/1 and TLR2/6 heterodimers, with comparable dose-response curves.

**Figure 3:**
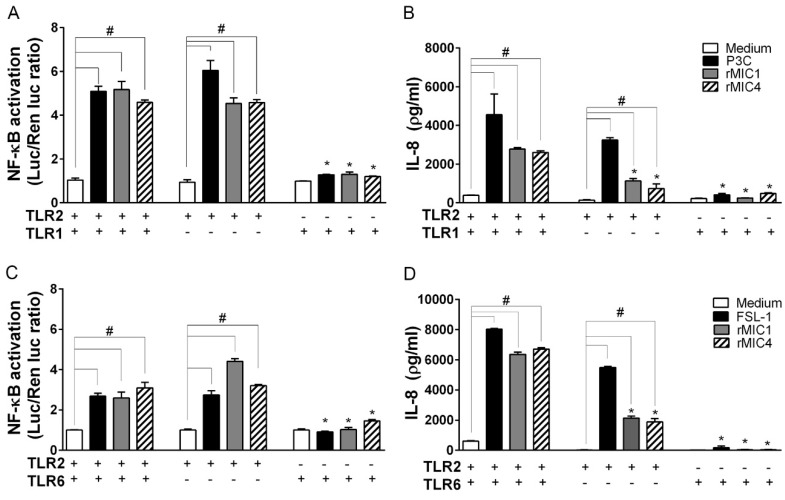
TLR2 is critical for cell activation stimulated by microneme proteins. HEK293T cells were transfected with TLR2/1, only TLR1, or only TLR2 (**A**,**B**) or with TLR2/6, only TLR6, or only TLR2 (**C**,**D**). The cells were also co-transfected with the co-receptors CD14 and CD36, the NF-κB-dependent luciferase reporter construct (pELAM-firefly luciferase), and a Renilla luciferase reporter construct (an internal control). The total amount of DNA in each transfection was kept constant by adding empty expression vector. After 48 h of transfection, the cells were stimulated with rMIC1 (50 nM) or rMIC4 (50 nM). The positive controls for cell stimulus were Pam3CSK4 (P3C, 1 nM) for TLR2/1-transfected cells and FSL-1 (1 nM) for TLR2/6-transfected cells. Medium was used as the negative control. Cells were lysed 4 h after stimulation, and NF-κB activation was inferred from the luminescence measurements. Cell supernatants were harvested 24 h after stimulation, and the IL-8 concentration was assessed by ELISA. (+) Cells expressing the receptor and (-) cells without expression of receptor. In the statistical analysis, the responses of (*) cells lacking a receptor were compared to those of cells expressing heterodimers (TLR2/1 or TLR2/6). In addition, in cells expressing combinations of receptors or a single receptor, statistical comparisons (#) of responses the elicited by rMIC proteins versus the responses in the negative control (medium) were also performed. The data are from three independent experiments yielding similar results. (*) or (#) *p* < 0.05 by one-way ANOVA followed by Bonferroni’s test.

We investigated the importance of TLR2 heterodimerization for cell activation induced by microneme proteins by comparing the responses of TLR2-transfected HEK293T cells with and without TLR1 (Figure 3A,B) or TLR6 (Figure 3C,D). Stimulation of cells expressing only TLR2 significantly increased IL-8 production to levels higher than those in the negative controls (medium, unstimulated cells), although the levels were not as high as those in cells transfected with TLR2/TLR1 or TLR2/TLR6 heterodimers. NF-κB activation induced by rMICs reached similar intensities in both TLR2 heterodimer-transfected cells and in cells expressing TLR2 only (Figure 3A,C). In contrast, cells transfected with only TLR1 or TLR6 (i.e., without TLR2) did not respond to the rMICs. These results show that heterodimerization of TLR2 with TLR1 or TLR6 enhances cell activation, and only the absence of TLR2 blocks IL-8 production stimulated by microneme proteins. Therefore, TLR2 is critical for cell activation induced by rMIC1 or rMIC4.

**Figure 4:**
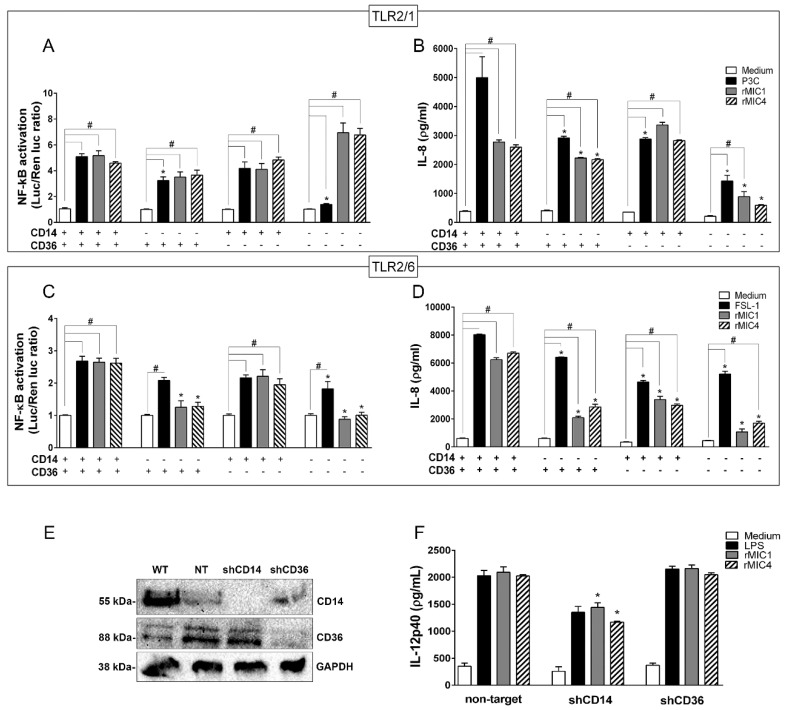
The CD14 and CD36 co-receptors amplify TLR2-mediated cell activation induced by microneme proteins. HEK293T cells transfected with (**A**,**B**) TLR2/1 or (**C**,**D**) TLR2/6 were co-transfected with CD14 and CD36 either together or alone, along with an NF-κB-dependent luciferase reporter construct (pELAM-firefly luciferase) and a Renilla luciferase reporter construct (an internal control). The total amount of DNA in each transfection was kept constant by adding empty vector. After 48 h of transfection, the cells were stimulated with rMIC1 (50 nM) or rMIC4 (50 nM). Pam3CSK4 (P3C, 1 nM) was used as a positive control for TLR2/1, and FSL-1 (1 nM) was used as a positive control for TLR2/6. Medium was used as the negative control. Twenty-four hours post-stimulation, the IL-8 concentration in cell supernatants were assessed by ELISA. Statistical analysis was performed to compare (*) the responses in cells lacking one or more co-receptor to the responses when both CD14 and CD36 were expressed. The responses in cells expressing one co-receptor, both co-receptors, or no co-receptor (#) were compared after stimulation with microneme protein versus the responses in the negative control cells (medium). The data are from three independent experiments yielding similar results (**E**) BMDMs obtained from wild type C57BL/6 mice were transduced with lentivirus vectors encoding shRNA sequences for CD14 and CD36 or a non-target control shRNA. The expression levels of CD14 and CD36 were evaluated by immunoblotting. (**F**) BMDMs deficient in CD14 or CD36 were stimulated with rMIC1 (5 µg/mL) or rMIC4 (5 µg/mL). LPS (1 µg/mL) was used as a positive control, and medium was used as a negative control. The IL-12 concentration in cell supernatants was measured by ELISA. (*) Statistical analysis of the responses in CD14- or CD36-knockdown cells versus that in non-targeted shRNA-transduced cells. (*) or (#) *p* < 0.05 by one-way ANOVA. Data from two independent experiments yielding similar results.

Because TLR2-mediated cell activation induced by various stimuli frequently engages CD14 and CD36, we asked whether rMIC1- and rMIC4-stimulated cell responses require these co-receptors. Figure 4A shows that HEK293T cells expressing TLR2/1 and CD14 and/or CD36 activate NF-κB in response to microneme proteins and the control agonist P3C. Interestingly, HEK293T cells expressing TLR2/1 but neither of the co-receptors (CD14 and CD36) showed significantly lower activation when induced with P3C but not with the microneme proteins. IL-8 production induced by rMIC1 and rMIC4 was measured in the supernatant of the same cells (Figure 4B), which showed reduced production in the absence of CD14 or either co-receptor (CD14 or CD36); a similar pattern was observed in cells stimulated with P3C. In contrast to the observation in microneme protein-stimulated cells, cells stimulated with P3C required CD36 to produce IL-8. In HEK293T cells expressing TLR2/6 (Figure 4C), NF-κB activation induced by both microneme proteins was markedly reduced in the absence of CD14 and either co-receptor (CD14 or CD36), whereas the response to the positive control agonist, FSL-1, was only slightly reduced in the absence of co-receptor. rMIC-induced IL-8 production in cells expressing TLR2/6 (Figure 4D) was also diminished in all conditions of co-receptor absence but was only slightly reduced in FSL-1-stimulated cells. However, IL-8 production did not decline to basal levels (in unstimulated cells) under any tested experimental condition. A second approach was employed to investigate whether the results obtained with HEK293T lineage cells could be duplicated in BMDMs, specifically regarding the dependence of rMIC-stimulated responses on co-receptors. We silenced CD14 and CD36 expression in BMDMs by transducing the cells with a lentiviral vector encoding shRNAs targeting CD14 or CD36, or a non-target shRNA as a control. Immunoblotting analysis demonstrated the efficiency of co-receptor knockdown (Figure 4E). Compared to cells transduced with the non-target shRNA, CD14-deficient BMDMs stimulated with rMIC1 or rMIC4 showed decreased IL-12 production, and this response not observed in CD36-deficient cells (Figure 4F). Taken together, our results demonstrate that in the receptor complex, TLR2 is crucial for rMIC-induced cell responses, i.e., NF-κB activation and cytokine production, and TLR2 heterodimerization and co-receptors engagement amplify these responses.

## 3. Discussion

In this study, we described new aspects of a recently described role for two lectins from *T. gondii*, MIC1 and MIC4. These microneme proteins were previously shown to be involved in the adhesion and invasion of host immune cells, and they are known to interact with TLR2 N-glycans on host immune cells and promote pro-inflammatory cytokine production, both of which favor host protection in the early phases of infection. *T. gondii* tachyzoites preassemble their microneme proteins in the endoplasmic reticulum and are then transported to and stored in apical microneme organelles [35]. These microneme proteins form complexes, such as the well-studied complex consisting of MIC6 [7], a membrane-spanning protein, MIC1 [14,36,37], and MIC4 [7,14,38] two soluble adhesions. The MIC1/4/6 complex contributes host cell adhesion and invasion. MIC1 has been shown to contribute to parasite virulence in mice [36]. However, disruption of *MIC1* and *MIC4* did not affect parasite survival [7] nor host cell attachment [36].

The propensity of microneme proteins to form oligomers with several other protein partners expands the repertoire of host targets for parasite binding [14]. The lectin domains of MIC1 and MIC4 specifically recognize α2-3-sialyllactosamine [8,13,16] and β1-3- or β1-4-galactosamine [6,14,16], respectively, which are often found at the terminal positions of glycans on the surface of mammalian cells. The diversity and complexity of glycan structures found on these cells make lectins available for numerous biological and pathological process, such as adherence, invasion, and colonization [37,39,40,41,42].

Recently, MIC1 and MIC4 were shown to function as immunomodulatory agents that drive the host response toward the protective T helper 1 axis (Th1) [16,17,18]. Th1 cell activation leads to the production of IL-12, a cytokine that induces T and NK cells to release IFN-γ, which is a trademark of Th1 responses and plays a pivotal role in host protection against intracellular pathogens [29,43,44]. This MIC1- and MIC4-triggered activity is driven by TLRs, which activate signaling pathways to induce pro-inflammatory cytokine production by macrophages and dendritic cells [16]. In addition, we showed that immunization with MIC1 and MIC4 confers efficient protective immunity against *T. gondii* infection [17,18]. A similar effect against *T. gondii* infection was observed following immunization with the microneme proteins MIC3, MIC6, MIC8, MIC11, and MIC13 [45,46,47,48,49].

The TLR ectodomains contain leucine-rich repeats (LRR) intercalated with different amino acid sequences, which lead to variable conformational structures in these domains [20]. This structural variability, combined with heterodimer formation and co-receptor engagement, provide an extended platform in the TLR architecture for the recognition of varied sets of ligands [20,50,51]. The TLR2 and TLR4 ectodomains contain four and nine N-linked glycans, respectively [52]. N-glycans linked to receptors, such as TLRs, support protein biosynthesis and transport to the cell membrane [52,53] and provide strategic targets for lectins. Glycan-lectin interactions may initiate receptor activation [16,54,55,56]. The interaction of the rMIC1 and rMIC4 lectin domains with glycans N-linked to the ectodomains of TLR2 and TLR4 on host macrophages and dendritic cells induces high IL-12 production [16].

Interaction of TLR with PAMPs induces the dimerization of the receptor ectodomains and consequent conformational changes that allow the self-association of cytosolic, toll-like interleukin receptor-1 (TIR) domains, providing scaffolds for downstream signaling [57]. This process culminates in NF-κB activation and transcription of pro-inflammatory cytokines, chemokines, and type I interferons [20]. Here, we showed that stimulation of RAW264.7-*luc* macrophages with either rMIC1, rMIC4, or Lac+ promotes NF-κB activation, similar to that induced by PAMPs. Remarkably, transfection of HEK293T cells with TLR2 reproduces this NF-κB response, an indication that the interaction of rMIC1 or rMIC4 with TLR2 alone is sufficient to trigger cell activation.

The binding of rMIC1 or rMIC4 to TLR2 homo- or heterodimers promotes Myddosome formation. Myd88 recruits IL-1R-associated kinase protein-1 (IRAK-1), IRAK-4, and TNF receptor-associated factor 6 (TRAF6) [57]. IRAK-1 mediates the ubiquitination of TRAF6, which, in turn, activates TGF-β activated kinase 1 (TAK1) [58]. TAK1 activation leads to two different pathways: NF-κB and MAPK, and both are dependent on the IKK kinase (IKK) complex, which consist of catalytic subunits (IKKα and IKKβ) and regulatory subunits (IKKγ). The IKK complex induces degradation of the NF-κB inhibitor IκBα and other IκB family members, such as p105. These events result in the release and nuclear translocation of canonical NF-κB family members, predominantly NF-κB1 p50–RELA and NF-κB1 p50–c-REL dimers, which is followed by the induction of pro-inflammatory gene expression [59,60,61]. TAK1 also mediates the activation of MAPK family members, such as ERK1/2, p38, and JNK, which activate AP-1 family transcription factors [62]. Activation of p38 downstream of TAK1 and transcription of NF-κB are essential for cell activation in response to microneme protein stimulus. Because TAK1 is a central component of the canonical NF-κB and MAPK signaling pathways [63], its activity is necessary for IL-12 up-regulation induced by rMIC1 and rMIC4. Although non-canonical NF-κB activation was not experimentally excluded, IL-12 production was abolished in BMDMs pre-treated with a TAK1 inhibitor and stimulated with microneme protein, indicating that non-canonical activation is not relevant to the cell response to rMIC1 or rMIC4.

As mentioned above, a small number of *T. gondii* components are known to activate cell signaling in a TLR-dependent manner. However, no study has attributed this activation to carbohydrate recognition [23,24,25,26,27,28,29,64]. Despite significant progress in understanding how TLRs function during *T. gondii* infection, the molecular mechanisms underlying parasite recognition are still unclear. This study demonstrated that even nanomolar concentrations of rMIC1 or rMIC4 activate TLR2/1 and TLR2/6 heterodimers. The response of cells expressing TLR2 heterodimers to increasing concentrations of rMIC1 and rMIC4 followed a bell-shaped curve, as was reported in studies of plant lectins, such as ArtinM, Euphorbin, *Viscum album* agglutinin I, and garlic lectin [55,65,66,67,68,69], and pathogen lectins, such as Paracoccin [54]. Similar bell-shaped dose-response curves could probably be drawn for rMIC1 if a broader range of concentrations were tested.

Although TLR2 heterodimers are not required for cell activation, when compared to the response of TLR2 alone, the TLR2 heterodimers amplified cell activation. This conclusion is supported by the demonstration that HEK cells transfected with TLR2 alone respond to rMIC and is reinforced by a previous report showing that the interaction of *Mycobacterium leprae* with TLR2 alone or with TLR2/1 heterodimers mediates cell activation [70]. Agonist binding to TLR ectodomains results in the formation of receptor dimers that are capable of recognizing a more substantial range of ligands [30]. The existence of TLR heterodimers is well supported in the literature, including the following combinations: TLR2/1 [71], TLR2/6 [72], and TLR4/6 [73]. Homodimers have also been reported, including TLR2/2 [70], TLR3/3 [74], TLR4/4 [75], and TLR5/5 [76]. In addition, TLR2 complexes on the cell surface are known to interact with the co-receptors CD14 [77,78,79] and CD36 [31,80,81], forming three different heterocomplexes: (1) The CD14-TLR2-TLR1 complex, which interacts with triacylated lipoproteins [78,81] and microbial components, such as Salmonella curli fibers [82]; (2) the CD36-CD14-TLR2-TLR1 complex, which recognizes lipomannan/ lipoarabinomannan from Mycobacteria [83,84]; and (3) the CD36-CD14-TLR2-TLR6 complex, which binds lipoteichoic acid (LTA) and diacylated lipoproteins [50,81]. All of the previously mentioned interactions were reported to induce cell activation. Our group previously verified that macrophages stimulated with ArtinM, a lectin that recognizes the trimannoside core of N-glycans, induces IL-12 production, in a manner that requires binding to both TLR2 and CD14 N-glycans. This membrane complex is a sensitive target for cell activation initiated by carbohydrate recognition [55,85].

Our results demonstrate that CD14 and CD36 contribute to, but are not required for, TLR2/1 or TLR2/6 activation stimulated by rMIC1 or rMIC4. In the absence of either co-receptor (CD14 or CD36), TLR2/1- and TLR2/6-transfected HEK293T cells responded to the microneme proteins by inducing significant NF-κB activation and IL-8 production, although the IL-8 levels were relatively low. Consistently, CD14- and CD36-silenced macrophages produced significant levels of IL-12 in response to stimulation with either microneme protein. Presumably, these variable reductions in the responses are due to the distinct abilities of the TLR2 complexes to trigger the activation of more than one signaling pathway [86], indicating that the mechanisms underlying signaling pathway activation by the TLR complexes deserve further investigation. Regarding potential polymorphisms within the TLR 1, 2, 4, 6 genes or their co-receptors, there is no data relating these genetic alterations to N-glycan(s) loss, which could be relevant for the recognition by MICs. Briefly, the results reported herein indicate that TLR2/TLR1 and TLR2/TLR6 heterodimerization and engagement of the co-receptors CD36 and CD14 enhance cellular activation in response to microneme protein stimulation but are not critical for activation. Indeed, in the receptor complex, only TLR2 was critical for cell activation induced by rMIC1 or rMIC4. These data add new information to our current understanding of the relationship between *T. gondii* and the innate host defense.

During infection, *T. gondii* employs complex strategies to modulate host immunity. Innate immune cells frequently use lectin-carbohydrate interactions to capture and destroy pathogens and/or to trigger an appropriate response to pathogens [87,88]. Microneme proteins trigger TLR2 function, which is enhanced by receptor heterodimerization and co-receptor engagement. These events delineate a model for a particular host-parasite interaction. In this model, *T. gondii* lectins adhere the parasite to the surface of most host cells via glycoconjugate recognition, supporting successful cell invasion. Because the range of interactions established by microneme lectins includes binding to TLR N-glycans, the recruitment of additional molecules to the TLR complex helps drive the development of a pattern of acquired immune responses against the parasite. In this sense, the binding of microneme lectins to TLR on phagocytes favors effective host defense. Considering how advantageous the triggered events are for host protection, MIC/TLR interactions deserve to be carefully dissected, with an aim to design new strategies to treat *T. gondii* infection. It may be possible to construct structures that mimic the sugar-binding property of MIC1 and MIC4. This is not a simple task, especially considering the fact that carbohydrate recognition occurs in water, making it necessary to maintain the hydromimetic exterior of the carbohydrates [89,90,91]. To be functional, synthetic analogs of MIC1 and MIC4 should bind to the terminal sialic acid or galactose residues of innate immune receptors in an aqueous environment. Once obtained, these molecules would be valuable for suppressing the acute phase of toxoplasmosis and allowing the development of an appropriate acquired immune response that favors host protection.

The present study, combined with previous reports on the roles of MIC1 and MIC4 in the acute phase of *T. gondii* infection [16,17,18], define the role of TLR2 in the activation of host immune cells during parasite invasion. Moreover, the study provides new perspectives for the design of immunotherapeutic strategies to combat acute toxoplasmosis in susceptible hosts.

## 4. Materials and Methods

### 4.1. Animal Care and Ethics Statement

All animal procedures were performed in accordance with the Guide for the Care and Use of Laboratory Animals of the National Research Council and were approved by the Committee on Ethics in the Use of Animals of Ribeirão Preto Medical School, University of Sao Paulo (CEUA-FMRP-USP—protocol numbers 191/2017 from 09/11/2017 and 065/2012 from 06/29/2012). Female, 8–12-week-old, C57BL/6 mice weighing 20–25 g (*n* = 5) were obtained from the University of São Paulo—Ribeirão Preto campus animal facility (Ribeirão Preto, São Paulo, Brazil) and housed in the animal facility of the Department of Cell and Molecular Biology, Ribeirão Preto Medical School, University of São Paulo under specific pathogen-free conditions. The mice were acclimated to the facility for 1 week before starting the experiment and were housed in individual ventilated cages in light-tight cabinets (Alesco, Capivari, Brazil), and maintained at 20–22 °C under a 12-h light-dark cycle and given ad libitum access to chow and water. All cages were bedded with autoclaved softwood shavings and cleaned twice a week.

### 4.2. Preparation of the Lac+ Fraction from T. gondii and Recombinant MIC1 and MIC4

The lactose-bound fraction (Lac+) was obtained from *T. gondii* as previously reported [12,18]. Briefly, a preparation of soluble tachyzoite antigens (STAg) was loaded into a lactose-agarose column (Sigma-Aldrich, St. Louis, MO, USA) equilibrated with PBS containing 0.5 M NaCl. The material adsorbed to the resin was eluted with 0.1 M lactose in equilibrating buffer and dialyzed against ultrapure water. The lactose-bound fraction was denoted Lac+, and the presence of MIC1 and MIC4 in the fraction was confirmed. Cloning, expression, and refolding of the recombinant 6-histidine-tagged microneme proteins MIC1 and MIC4 were carried out as described previously [18]. All preparations were examined for endotoxin contamination using the Limulus Amebocyte Lysate Kit (QCL-1000; Lonza, Basel, Switzerland). The preparations of rMIC1 and rMIC4 contained 7.2 and 1.1 EU of endotoxin/µg protein, respectively. To further reduce the already low endotoxin concentrations, the preparations were applied to and eluted from polymyxin-B columns (Affi-Prep^®^ Polymyxin Resin; Bio-Rad, Hercules, CA, USA). Prior to their use in cell-stimulation experiments, aliquots of the preparations were incubated with 50 µg/mL polymyxin B sulfate salt (Sigma-Aldrich) for 30 min at 37 °C to neutralize any residual endotoxin.

### 4.3. RAW264.7-Luc Cell Culture

RAW264.7-*luc* cells, a mouse macrophage cell line with an NF-κB promoter-luciferase construct (pNF-κB-Luc), were kindly provided by Dr. Dario Zamboni and were cultured in Dulbecco’s modified Eagle’s medium (DMEM) supplemented with 10% heat-inactivated fetal bovine serum (FBS), 10 U/mL penicillin, and 10 µg/mL streptomycin (Gibco, Thermo Fisher Scientific, Inc., Grand Island, NY, USA). Cultures were maintained at 37 °C in a 5% CO_2_ humidified atmosphere, and the experiments were perform on cells at 60–70% confluence. Cells were stimulated with rMIC1, rMIC4, or Lac+ (10 µg, each). Cells incubated with 100 ng/mL Ultrapure LPS (standard LPS, *E. coli* 0111:B4; Sigma-Aldrich) or medium were used as the positive and negative controls, respectively. After 20 h, the cells were lysed for the luciferase reporter assay.

### 4.4. Preparation of Bone Marrow-Derived Macrophages

Bone marrow-derived macrophages (BMDMs) were obtained from C57BL/6 mice as previously described [34]. Briefly, bone marrow was harvested from the femurs and hind leg bones. After washing with RPMI medium, the cells were resuspended in RPMI medium supplemented with 10% FBS, 10 U/mL penicillin, and 10 µg/mL streptomycin (Gibco). For macrophage differentiation, 30% L929 conditioned medium was added to RPMI medium supplemented with 10% FBS. Cells were cultured in 100 × 20 mm dishes (Costar; Corning, Inc., Corning, NY, USA) for 7 days; the conditioned medium was added at day 4. On day 7, non-adherent cells were removed and analyzed by flow cytometry to determine their macrophage phenotype. Over 92% of the harvested cells expressed high levels of F4/80 antigen.

### 4.5. Cell Signaling Inhibition Assay

BMDMs were harvested from C57BL/6 mice, plated into 24-well plates at 5 × 10^5^ cells/well, and incubated for 3 h with the following pharmacological inhibitors of MAP-kinases: TGF-β activated kinase 1 (TAK1), 5Z-7-oxozeaenol, 100 nM; extracellular-signal-regulated kinase (ERK), PD98059, 20 μM; c-Jun N-terminal kinase (JNK), SP600125, 20 μM; p38, SB202190, 20 μM; Ser and Thr kinase (AKT), Wortmannin, 100 nM; nuclear factor-kappa B (NF-κB), and caffeic acid, 15 µg/mL (Sigma-Aldrich). Cells were stimulated with rMIC1 or rMIC4 (5 μg/mL) for 24 h. LPS (100 ng/mL) and medium were used as the positive and negative controls, respectively. Staurosporine (2 µM) and Triton X-100 (1% *v*/*v*) were used as a positive control for in vitro apoptosis. The concentration of IL-12p40 in the cell culture supernatants was determined using a standard ELISA. Cell viability was examined after treatment with Alamar Blue (Invitrogen, Carlsbad, CA, USA; 1/10 dilution) at 18, 24, and 48 h. Fluorescence measurements were performed on an FLx800 Fluorescence Microplate Reader (BioTek Instruments, Winooski, VT, USA; excitation, 590 nm; emission, 635 nm).

### 4.6. Western Blotting

To evaluate p38 and NF-κB phosphorylation, 1 × 10^7^ BMDMs were treated with rMIC1 (5 µg/mL), rMIC4 (5 µg/mL), or LPS (100 ng/mL) for 24 h. Then, the cells were lysed in a buffer containing 100 mM NaCl, 20 mM Tris (pH 7.6), 10 mM EDTA (pH 8), 0.5% SDS, and 1% Triton X-100 with protease inhibitor cocktail (Sigma-Aldrich) and incubated for 20 min on ice. Laemmli sample buffer was added to the lysates, and the samples were boiled for 10 min. Proteins were then separated by SDS-PAGE on 10% polyacrylamide resolving gels and transferred to nitrocellulose membranes. The primary antibodies used were: Phospho-NF-κB p65 (Ser536, 93H1, 1:1000; Cell Signaling Denvers, MA, USA; cat. number 3033), NF-κB p50 (E-10) mouse (1:500; Santa Cruz, Dallas, TX, USA; cat number sc-8414), phospho-p38 MAPK (Thr180/Tyr182, 28B10, 1:100; Cell Signaling; cat. number 9216), p38 MAPK (1:1000; Cell Signaling; cat. number 9212), and glyceraldehyde-3-phosphate dehydrogenase (1:2000; Trevigen, Gaithersburg, MD, USA; cat. number 2275).

### 4.7. Transfection of HEK293T Cells with TLR2 Complexes

Human embryonic kidney 293T (HEK293T) cells, originally acquired from the American Tissue Culture Collection (ATCC, Rockville, MD, USA), were cultured in DMEM, supplemented with 10% FBS (Gibco) at 37 °C in a humidified atmosphere of 5% CO_2_. The day before transfection, HEK293T cells were seeded in 12-well plates (5 × 10^5^ cells/ well). Cells (at 70–80% confluence) were then transiently co-transfected with mouse TLR2, a combination of TLR2 and TLR1 (TLR2/1), or TLR2 and TLR6 (TLR2/6), and CD14 and CD36. Cells used for the luciferase reporter assays were also co-transfected with the NF-κB-dependent pELAM-luciferase reporter gene construct and the internal control Renilla plasmid as described previously [92]. Transfections were performed using Lipofectamine 2000 (Invitrogen) according to the manufacturer’s recommendations. The amount of transfected DNA per well was normalized to 2 µg by adding empty vector. All plasmids used for transfection were purified using the EndoFree plasmid kit (Qiagen, Chatsworth, CA, USA). After 24 h, the transfected cells were transferred to 96-well plates (4 × 10^4^ cells/well). After an additional 24 h, the cells were stimulated with Lac+, rMIC1, or rMIC4. Medium was used as a negative control for cell stimulation. The positive controls were Pam3CSK4 (P3C; EMC Microcollections, Baden-Württemberg, Tübingen, DEU), which is an agonist of TLR2/1, and fibroblast stimulating ligand-1 (FSL-1; EMC Microcollections) or macrophage-activating lipopeptide-2 (MALP-2; EMC Microcollections), which are agonists of TLR2/6. The stimulation period was 4 h for the luciferase reporter assay or 24 h for IL-8 detection, the most inducible cytokine in HEK293 cells [93,94,95]. Cells transfected with empty vectors and stimulated with either medium or agonist (FSL-1 or P3C) were also analyzed. The absence of *Mycoplasma* contamination in the cell cultures was certified by indirect fluorescence staining, as described previously [94].

### 4.8. Luciferase Reporter Assays

Cells transfected with TLR2 complexes and co-transfected with the NF-κB-dependent promoter (ELAM-1-firefly luciferase) and a Renilla luciferase reporter construct (β-actin-Renilla luciferase) were analyzed using the Dual-luciferase Reporter Assay System (Promega, Madison, WI, USA), according to the manufacturer’s instructions. After stimulation as described above, cells were washed once in PBS and lysed using Passive Lysis Buffer (Promega). NF-κB-dependent firefly luciferase (FL) and constitutively expressed Renilla luciferase (RL) activities were measured using a Sinergy 2 luminometer (BioTek) and recorded in relative luminescence units (RLU). Luciferase activity was reported as the ratio of FL to RL. RAW264.7*-luc* cells were similarly treated, and the results are reported in relative luminescence units (RLU). All values were normalized to the relative luminescence from unstimulated (medium only) control transfected cells.

### 4.9. Cd14 and Cd36 Knockdown by shRNA

Recombinant viral particles were obtained by transfecting HEK293 cells with the helper plasmids psPAX2 and pMDG.2 and plasmids encoding either specific Cd14 or Cd36 shRNA sequences or control (non-target) shRNA. The shRNA sequences used were as follows: CD14, 5′- CCGGCCTTGTGAGCTGGACGATGAACTCGAGTTCATCGTCCAGCTCACAAGGTTTTT-3′ and CD36, 5′-CCGGCGGATCTGAAATCGACCTTAACTCGAGTTAAGGTCGATTTCAGATCCGTTTTTG-3′. Supernatant containing lentivirus was added to cultured bone marrow cells that had been incubated for 3 days with 10% L-cell conditioned medium (LCCM). The cells were incubated overnight at 37 °C in 5% CO_2_. The supernatant was then replaced with fresh medium supplemented with puromycin (3 µg/mL), and the cells were incubated for an additional 48 h. CD14- and CD36-knockdown BMDMs were harvested and seeded into 96-well plates at 1 × 10^6^ cells/mL for rMIC1, rMIC4, or LPS stimulation, followed by ELISA. CD14 and CD36 expression was systematically evaluated by immunoblotting. The primary antibodies were as follows: CD14 (F09, 1:500; Santa Cruz Biotechnology; cat. number sc-73794), CD36 (1:500; Thermo Fisher Scientific; cat. Number PA5-27236), and glyceraldehyde-3-phosphate dehydrogenase (GAPDH, 1:2000; Trevigen; cat. Number 2275-PC-020).

### 4.10. Measurement of Cytokines

The concentrations of mouse IL-12p40 and human IL-8 in the cell culture supernatants were determined by ELISA (OptEIA set; BD Biosciences, San Jose, CA, USA) in accordance with the manufacturer’s instructions. Appropriate recombinant cytokines were used to generate standard curves and determine the respective cytokine concentrations in the supernatant samples. The absorbance at 450 nm was measured on a Power Wave-X spectrophotometer (BioTek Instruments, Inc.).

### 4.11. Statistical Analysis

Statistical analysis of data was performed by one-way analysis of variance (ANOVA) followed by Bonferroni’s multiple comparisons. Results are presented as the mean ± standard deviation (SD). Differences were considered significant when p values were less than 0.05. Data were analyzed using GraphPad Prism software (GraphPad Prism version 6.00 for Windows, La Jolla, CA, USA).

## 5. Conclusions

The current study, in which we dissected the interactions established by rMIC1 and rMIC4, yielded the first evidence that these proteins activate the same signaling pathways activated by typical lipopeptide agonists. In addition, we showed that TLR2 heterodimerization and the co-receptors CD14 and/or CD36 augment, but are not required for, cell activation in response to rMIC1 and rMIC4. Therefore, among the constituents of the TLR2 complex, the interaction of rMIC1 and rMIC4 with the N-glycans of TLR2 is sufficient to induce cytokine production by innate immune cells.

## Supplementary Materials

Supplementary materials can be found at https://www.mdpi.com/1422-0067/20/20/5001/s1.
