# Supplementary material for: Receptor Heterodimerization and Co-Receptor Engagement in TLR2 Activation Induced by MIC1 and MIC4 from Toxoplasma gondii
Source: Int J Mol Sci. 2019 Oct 10;20(20):5001. doi: 10.3390/ijms20205001 (PMC6829480; doi:10.3390/ijms20205001)
Supplement: Supplementary file 1 [file ijms-20-05001-s001.pdf]

Receptor Heterodimerization and Co-receptor Engagement in TLR2 Activation Induced by MIC1 and MIC4 from *Toxoplasma Gondii*

Flávia Costa Mendonça-Natividade, Carla Duque Lopes, Rafael Ricci-Azevedo, Aline Sardinha-Silva, Camila Figueiredo Pinzan, Ana Claudia Paiva Alegre-Maller, Lilian L. Nohara, Alan B. Carneiro, Ademilson Panunto-Castelo, Igor C. Almeida and Maria Cristina Roque-Barreira

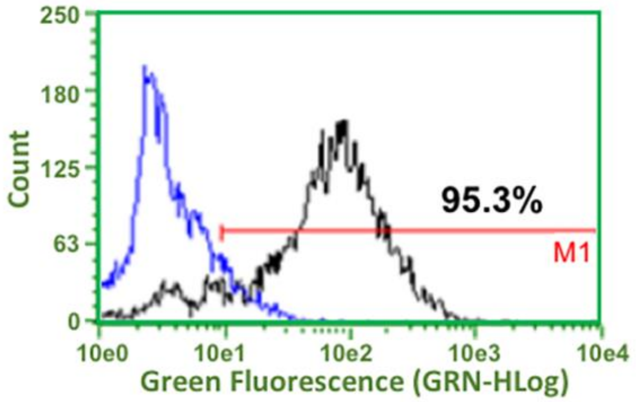

Figure S1. Bone marrow-derived macrophages obtained from bone marrow hematopoietic stem cells express F4/80 antigen. F4/80 is shown as a black line, and the isotype control is shown as a blue line. Graph represents the mean percentage of the mean fluorescence intensity (MFI).
